# Parameters for accurate genome alignment

**DOI:** 10.1186/1471-2105-11-80

**Published:** 2010-02-09

**Authors:** Martin C Frith, Michiaki Hamada, Paul Horton

**Affiliations:** 1 Computational Biology Research Center, Institute for Advanced Industrial Science and Technology, Tokyo 135-0064, Japan; 2 Mizuho Information & Research Institute, Inc. 2-3 Kanda-Nishikicho, Chiyoda-ku, Tokyo 101-8443, Japan

## Abstract

**Background:**

Genome sequence alignments form the basis of much research. Genome alignment depends on various mundane but critical choices, such as how to mask repeats and which score parameters to use. Surprisingly, there has been no large-scale assessment of these choices using real genomic data. Moreover, rigorous procedures to control the rate of spurious alignment have not been employed.

**Results:**

We have assessed 495 combinations of score parameters for alignment of animal, plant, and fungal genomes. As our gold-standard of accuracy, we used genome alignments implied by multiple alignments of proteins and of structural RNAs. We found the HOXD scoring schemes underlying alignments in the UCSC genome database to be far from optimal, and suggest better parameters. Higher values of the X-drop parameter are not always better. E-values accurately indicate the rate of spurious alignment, but only if tandem repeats are masked in a non-standard way. Finally, we show that γ-centroid (probabilistic) alignment can find highly reliable subsets of aligned bases.

**Conclusions:**

These results enable more accurate genome alignment, with reliability measures for local alignments and for individual aligned bases. This study was made possible by our new software, LAST, which can align vertebrate genomes in a few hours http://last.cbrc.jp/.

## Background

Genome sequence alignments are a priceless resource for finding functional elements (protein-coding sequences, RNA structures, *cis*-regulatory elements, miRNA target sites, etc.) and charting evolutionary history [1-4]. Many genome alignment algorithms have been developed, e.g. reviewed by [5]. All of these algorithms require selection of various mundane but critical parameters. In the most classic approach to alignment (Smith-Waterman/BLAST), these parameters include the scoring matrix and gap costs, which determine alignment scores, and thus which alignments are produced. This study aims to reveal the influence of these and other parameters, and to guide their selection for accurate genome alignment. Specifically, we investigate the following six facets of genome alignment:

### Alignment score cutoff

In the classic alignment framework, it is necessary to choose an alignment score cutoff: low enough to find weak homologies, but high enough to avoid too many spurious alignments. A rational approach is to calculate the E-value--the expected number of alignments between two random sequences scoring above the cutoff--and choose a cutoff that has an acceptable E-value. Surprisingly, this approach does not seem to be used for genome alignment (or if it is, it is not mentioned in method descriptions). The authors of BLASTZ tested their score cutoff by aligning two genomes after reversing, but not complementing, one of them [6]. Homology between reversed and non-reversed DNA is (thought to be) impossible, so this is a good measure of the spurious alignment rate, but it is inconvenient to repeat it with each new pair of genomes.

We have calculated the E-values implicitly used for several alignments in the UCSC genome database [7] (Additional file 1, Table S1). They vary between 5e-10 (human/chicken) and 14000 (*melanogaster*/*ananassae*). Often, higher E-value thresholds are used for genome alignment than would commonly be used for database searches (e.g. BLAST). This is reasonable because genome comparison produces many thousands of local alignments, and a few hundred or even a few thousand spurious alignments would only amount to a small fraction of these.

### Repeat-masking

There is a general awareness that repeat-masking is important for genome alignment, but the efficacy of repeat-masking methods has not been assessed in this context. "Repeats" can be categorized into two types: simple (low entropy) sequences such as ATATATATAT, and non-simple repeats such as *Alu *elements. Simple repeats cause spurious (i.e. non-homologous) alignments with high scores, but non-simple repeats do not, because e.g. every *Alu *is genuinely homologous to every other *Alu*. Non-simple repeats cause a different problem: too many alignments. In pursuit of accurate (homologous) alignment, we focus on simple repeats.

Many BLAST-like alignment tools have a capability known as "soft masking". This means that masking is applied for the first phase of the algorithm, when initial matches are found, but not for the second phase, when alignments are extended from the initial matches. This promises the best of both worlds: avoid purely repetitive alignments, but allow repeats within larger alignments.

### Scoring matrix

The scoring matrix specifies a score for aligning every kind of base with every other kind of base. The simplest scoring matrix, which is actually quite good for DNA, is: +1 for all matches and -1 for all mismatches. Given a set of trusted alignments, a scoring matrix is often derived using log likelihood ratios [8]. This is because, under simplifying independence assumptions, log likelihood ratio derived scores are theoretically optimal for discriminating between random and true alignments [9]. Unfortunately, real pairs of homologous sequences vary greatly in composition, and even more in conservation level; which means that the optimal matrix varies as well. To deal with this, matrices are sometimes constructed from alignments with low percent-identity, under the assumption that high percent-identity alignments will be found anyway [8]. Such matrices, however, will be worse at discriminating short alignments with high percent-identity from chance similarities [10,11]. Another approach is to develop a small number of compromise matrices that cover a range of percent-identities close-to-optimally [10,11]. A deeper problem is that, while log likelihood scores are optimal at distinguishing true from chance similarities (i.e. alignment-level accuracy), they are not necessarily optimal for accurate base-level alignment. Thus, although log likelihood ratios are useful to suggest features of scoring matrices, it is not self-evident that they will work best in practice. (For similar reasons, the Baum-Welch training algorithm [12] does not necessarily yield optimal alignment parameters for base-level accuracy.)

### Gap costs

Effective gap costs for protein alignment have been studied empirically [13], but not for DNA alignment.

### X-drop parameter

BLAST and similar methods, including BLASTZ and LAST, have an important but rarely considered X-drop parameter. (In NCBI BLAST and LAST this parameter is called "X", but in BLASTZ it is called "Y".) When extending gapped alignments, these methods terminate the extension when the score drops by more than X below the maximum previously seen [14]. This serves two purposes: to reduce computation time, and to prevent spurious internal regions in alignments. Without any X-drop criterion, maximum-score alignments can contain arbitrarily poor internal segments [15]. Thus the X-drop parameter is not merely an algorithmic detail: it is one of the parameters that define what a good alignment is. (These algorithms also have a second X-drop parameter used during the gapless alignment phase, but it does not constrain the final alignment, so we do not consider it here.)

### Base-level accuracy

Sequence alignments are inherently uncertain [16]. This uncertainty has traditionally been disregarded, leading to errors in many kinds of inferences made from alignments [17]. Fortunately, it is possible to quantify this uncertainty, by estimating an alignment probability for every possible pair of aligned bases [16,18]. We have made it easy to obtain these probabilities, by integrating their calculation into our alignment software, LAST. Below, we confirm that they are useful for measuring reliability and controlling the sensitivity/specificity tradeoff at the level of aligned bases.

### Previous work

Surprisingly, we could find only one publication that attempts to determine optimal score parameters for DNA alignment using actual genome data: that of Chiaromonte et al., which recommends the HOXD70 matrix [8]. This matrix has been adopted by several genome alignment tools [6,19,20]. That assessment, however, has several limitations: it used gapless alignment, it tested only nine scoring matrices, and it only used human-versus-mouse alignments. Most importantly, there are flaws in its evaluation procedure (see below). Thus, to date, there has been *no *rigorous assessment of DNA scoring schemes.

### Measuring alignment accuracy

It is notoriously difficult to measure the accuracy of genome alignments [6]. Some previous studies have used simulated alignments (e.g. [21]). Simulations are suitable for some tests, e.g. establishing the limits of various algorithms as in [16], but not for assessing alignment parameters, because the optimal alignment parameters will depend on the simulation parameters. Another study used mobile elements to assess noncoding DNA alignments, arguing that: "alignment of human *Alu *elements to any non-primate mammalian sequence is a false orthology prediction" [22]. This is not suitable for testing homology predictions, because, e.g. primate *Alu*s and rodent *B1 *elements both originate from retrotransposition of 7SL RNA, and are thus homologous [23]. (If their common ancestor did not duplicate before the divergence of primates and rodents, they would even be orthologous.)

We solve this problem only in a limited way, by using as gold-standards partial genome alignments implied by multiple alignments of proteins and of structural RNAs. Thus we measure alignment accuracy only in the parts of genomes that encode these molecules, and our conclusions might not apply to other parts of genomes. Our assessment is nevertheless useful. Although protein- and RNA-coding regions are thought to comprise only a small minority of large (e.g. mammalian) genomes, these regions are the focus of many downstream studies that use genome alignments. Moreover, small genomes (e.g. yeasts) consist mostly of coding sequence, as do the alignable parts of large but distantly related genomes (e.g. mammal versus non-mammal). Finally, we submit that there is little justification for genome alignment parameters used up till now, and a limited assessment is better than none.

Alignment accuracy can be measured at two levels: correctness of whole (local) alignments, or correctness of aligned bases. For genome alignment, the latter is arguably more relevant. This is because many downstream analyses, such as RNA structure prediction or detection of positively selected sites, depend on the base-level accuracy of the alignments [17]. In such analyses, more information is obtained from long alignments than from short alignments, making it inappropriate to weight the correctness of long and short alignments equally. This stands in contrast to protein database searches (e.g. BLAST), where residue-level accuracy is not always important, since we may wish to know only whether a protein is evolutionarily related to another protein. Unfortunately, the classic theory of alignment statistics has been developed from the standpoint of database searches and alignment-level accuracy [9].

This study does not address the problem of distinguishing orthologous from paralogous alignments. This important problem is very different from that of aligning homologous bases, and requires its own specialized methods.

In this study we empirically assess many parameter choices on whole genome alignments of several organisms. We were able to perform many thousands of genome alignments only by using our new alignment software, LAST. Our results give a practical guide for choosing repeat masking strategy, substitution and gap costs, and X-drop parameter - along with empirical estimates of false and true positive rates. We find that the best parameters significantly outperform current standard practice, often decreasing false positives by a factor of two or more at the same true positive rate.

## Results

### E-values and repeat-masking

**Figure 1:**
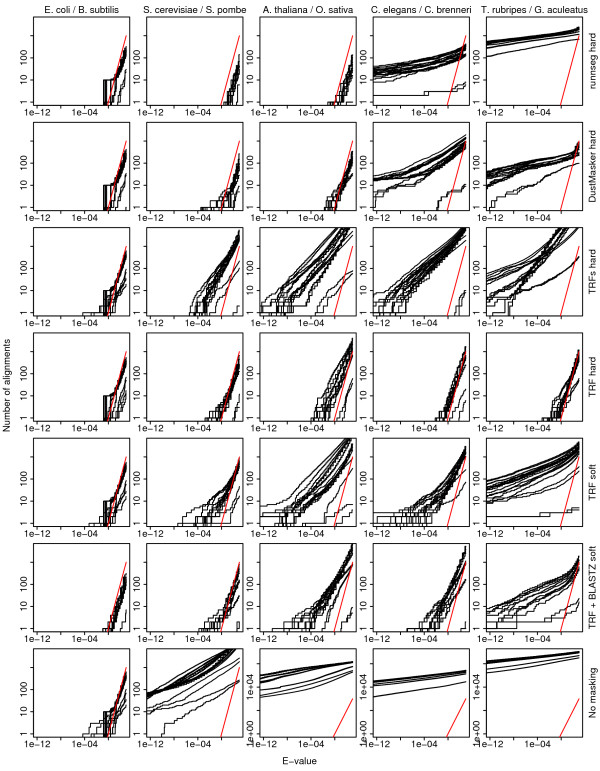
**E-values of reverse genome alignments with six repeat-masking methods**. In each column, the second-named genome was reversed and then aligned to the first-named genome twenty times, using twenty different scoring schemes. The red lines show the theoretically expected number of alignments at each E-value threshold, and the black lines show the observed number. Alignments in rows 1-5 and 7 were performed with LAST, and those in row 6 were done with BLASTZ, using BLASTZ's internal entropy-masking method. "TRFs": Tandem Repeats Finder with standard parameters; "TRF": Tandem Repeats Finder with non-standard parameters; "hard": hard-masking; "soft": soft-masking.

We tested E-value calculations and repeat-masking by aligning two genomes after reversing (but not complementing) one of them. Reversed genomes are convenient for estimating the rate of spurious alignments [6], because the reversed genome has the same composition and sequence complexity as the actual genome, but has no homology to any real genome. Figure 1 shows the results for five genome pairs and six repeat-masking methods [24-26]. For the bacterial genomes (left column), the observed numbers of alignments closely follow the theoretical E-value distribution, even with no masking. For the other genomes, with no masking, there are many spurious alignments with significant E-values (Figure 1, row 7). Since the alignment algorithm (LAST or BLASTZ) does not guarantee to find all high-scoring alignments, these results are lower bounds. Only one of the repeat-masking methods eliminates spurious alignments fairly effectively for all the genome pairs: TRF (Tandem Repeats Finder) with non-standard parameters and hard masking (Figure 1, row 4). It works well for mammalian genomes too (Additional file 1, Figure S1).

**Figure 2:**
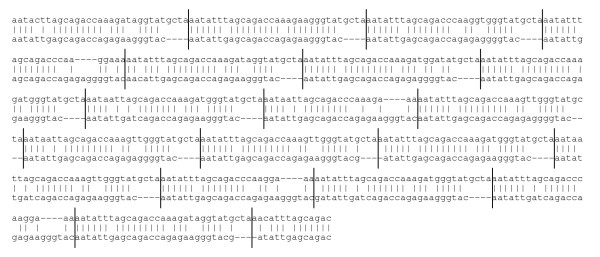
**A spurious similarity caused by tandem repeats**. The upper sequence is from the *C. elegans *genome and the lower sequence is from the reversed *C. brenneri *genome. DustMasker fails to mask these sequences.

Suppression of spurious alignments requires careful masking of tandem repeats. For example, Figure 2 shows a high-scoring, spurious alignment that DustMasker fails to eliminate. It is caused by tandem repeats: the repeat unit from *C. elegans *has a chance similarity to a similar-length reversed repeat unit from *C. brenneri*. Because these units are tandemly repeated, the total length of the similar sequence region is amplified - increasing the apparent statistical significance of the match. DustMasker and runnseg can detect short-period tandem repeats, but this example shows that it is important to detect longer-period repeats too.

We obtained the non-standard TRF parameters by trial and error: we simply lowered TRF's mismatch cost, gap cost, and score cutoff until it worked. It is likely that a more principled and effective repeat-masking method than this can be found.

**Figure 3:**
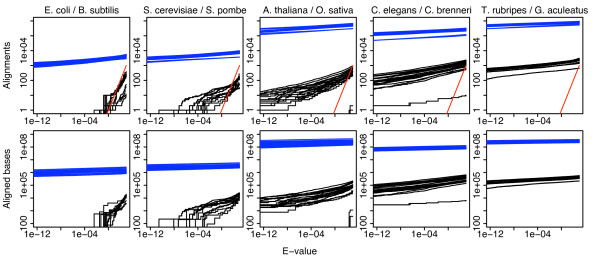
**Spurious alignment quantities compared to total alignment quantities, with soft repeat-masking**. The horizontal axis of each graph represents an E-value threshold and the vertical axis represents the number of alignments (first row) exceeding that threshold, and the number of aligned bases contained in those alignments (second row). In each column, the second-named genome was reversed (black lines) or not (blue lines), and then aligned to the first-named genome using twenty different scoring schemes. The red lines show the theoretically expected number of alignments at each E-value threshold. Repeat-masking was done with WindowMasker, including its DustMasker component.

In our tests, soft-masking fails to eliminate spurious alignments, but soft-masking *is *typically used for genome comparison, presumably out of reluctance to prevent any sequences from being aligned. Table 1 shows the proportions of genomes masked by several methods: typically under 10% for TRF. It might be argued that a certain level of spurious alignment is an acceptable price for using soft instead of hard masking. To examine this, Figure 3 compares quantities of spurious versus total alignment for a convenient soft-masking procedure (WindowMasker+DustMasker) [27]. With an appropriate score cutoff, the amount of spurious alignment is generally less than 1% of the amount of total alignment. However, if the E-value calculation is not reliable, it becomes harder to choose a rational score cutoff. Furthermore, it is not clear that tandem repeats can be meaningfully aligned even if they are known to be homologous. In summary, if our top priority is to avoid spurious alignments then we should use hard-masking, but if we are more concerned to align as much as possible then soft-masking may be appropriate.

**Table 1:** Percentages of genomes masked by four repeat-masking methods

Genome	**nseg**	**DustMasker**	**TRF**	**WindowMasker + DustMasker**
*T. rubripes*	8.8	6.6	6.1	19
*G. aculeatus*	9.0	6.6	5.6	22
*C. elegans*	11	15	10	37
*C. brenneri*	8.8	9.6	7.4	29
*A. thaliana*	11	9.6	6.5	23
*O. sativa*	10	9.6	8.8	33
*S. cerevisiae*	7.3	5.2	3.4	14
*S. pombe*	8.3	6.0	3.1	16
*E. coli*	2.5	1.1	1.3	7.5
*B. subtilis*	4.5	3.0	1.2	12

In the center column, DustMasker was run with option "-level 16".

### Assessment of scoring schemes

Here, we assess which score parameters give the most accurate alignments, with high sensitivity and specificity. In order to measure accuracy, we need "gold-standard" alignments. For gold-standards, we used genome alignments implied by multiple alignments of proteins in the TreeFam database and of structural RNAs in the Rfam database [28,29]. These gold-standards will not be perfectly correct, but they were constructed using considerably more information than pair-wise nucleotide similarity.

Table 2 (and Additional file 1, Table S2) shows log-likelihood-ratio scoring matrices derived from the gold-standard alignments, for several pairs of genomes. As explained above, they are not necessarily optimal in practice, but they provide clues. They are broadly similar to each other and to the HOXD70 matrix [8]. They all have lower penalties for transitions than transversions. The match scores reflect the composition: A and T matches receive lower scores than G and C matches for AT-rich alignments, and conversely for GC-rich alignments. The mismatch costs are weaker compared to the match scores for alignments with low percent-identity (e.g. the yeasts), and stronger for alignments with high percent-identity. Indeed the yeast matrix penalizes substitutions so weakly that it is prone to producing non-localized alignments. (With a gap existence cost of 400 and a gap extension cost of 30, the FLANK software [30] finds no limit to the extent of alignments of random sequences). Thus, we used these matrices only as a rough guide to design our parameter search.

**Table 2:** Log-likelihood-ratio scoring matrices

**Gg/Hs Rfam** **70% identity, 54% A+T**					**Tr/Hs Rfam** **66% identity, 53% A+T**					**At/Os Rfam** **69% identity, 55% A+T**				
		
	a	c	g	t		a	c	g	t		a	c	g	t
a	86	-110	-48	-94	a	87	-93	-49	-79	a	79	-92	-57	-69
c	-110	100	-118	-48	c	-93	100	-107	-49	c	-92	100	-117	-57
g	-48	-118	100	-110	g	-49	-107	100	-93	g	-57	-117	100	-92
t	-94	-48	-110	86	t	-79	-49	-93	87	t	-69	-57	-92	79
		
														
		
**Gg/Hs TreeFam** **65% identity, 49% A+T**					**Tr/Hs TreeFam** **57% identity, 46% A+T**					**Sc/Sp TreeFam** **49% identity, 60% A+T**				
		
	a	c	g	t		a	c	g	t		a	c	g	t
a	100	-99	-46	-113	a	100	-75	-38	-92	a	71	-55	-28	-60
c	-99	92	-82	-46	c	-75	82	-63	-38	c	-55	100	-58	-28
g	-46	-82	92	-99	g	-38	-63	82	-75	g	-28	-58	100	-55
t	-113	-46	-99	100	t	-92	-38	-75	100	t	-60	-28	-55	71

Scoring matrices derived from Rfam (top row) or TreeFam (bottom row) based gold standards for the indicated genome pairs are shown. Species names abbreviated as: Gg: *G. gallus*, Hs: *H. sapiens*, Tr: *T. rubripes*, At: *A. thaliana*, Os: *O. sativa*, Sc: *S. cerevisiae*, Sp: *S. pombe*. Additional file 1, Table S2 shows corresponding matrices for other genome pairs.

We tested alignment accuracy using 97 combinations of score parameters, which we denote in the format: match score: transition cost: transversion cost: gap existence cost: gap extension cost, e.g. "2:1:2:16:1". We also tested the BLASTZ/UCSC scoring schemes: the HOXD70 and HOXD55 matrices with a gap existence cost of 400 and a gap extension cost of 30. Each scoring scheme was combined with five X-drop values, for a total of 495 parameter combinations. In all cases, we hard-masked all the genomes with TRF, and aligned them with LAST using score cutoffs corresponding to an E-value of 1; an arbitrary choice. All genomes except the yeasts were additionally soft-masked with WindowMasker, to avoid wasting time on non-simple repeats. All parameters and results are tabulated in Additional file 2.

**Figure 4:**
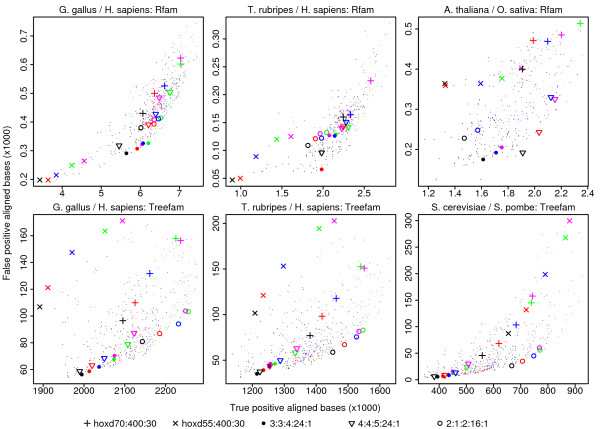
**Genome alignment accuracies with 495 combinations of score parameters**. Each point represents one genome alignment with one combination of score parameters. A few of these are highlighted with symbols: see the key beneath the figure. True positives (horizontal axis) and false positives (vertical axis) were counted with reference to either Rfam (upper row) or TreeFam (lower row). Colors indicate the X-drop parameter. For black points: the X-drop parameter was set to allow a maximum gap size of 20; red: 30; blue: 50; green: 100 and magenta: 200. The same results, but with different scoring schemes highlighted, are shown in Additional file 1, Figure S2.

Our test results are shown in Figure 4 (and Additional file 1, Figure S2). There are too many parameter combinations to show them all with distinct symbols, so we just highlighted a few interesting ones. (The same results on different genome pairs are shown in Additional file 1, Figures S3 and S4.) The tests using TreeFam as a gold-standard give very consistent results, even though we used genomes with different levels of similarity (e.g. human/chicken and human/pufferfish). The parameter combination marked with circles (2:1:2:16:1) gives an excellent balance of sensitivity and specificity, while HOXD55:400:30 is the worst of all those tested, and HOXD70:400:30 is mediocre. (Several matrices in Table 2 and Additional file 1, Table S2 have match: transition: transversion ratios close to 2:1:2, which fits with the good performance of 2:1:2:16:1.) The combination 1:1:1:7:1 is decent but conservative, and superior to 2:1:2:16:1 for the most closely-related genomes (*melanogaster*/*yakuba*, Additional file 1, Figure S4). The tests using Rfam as a gold-standard give less consistent results, but some trends are still evident. For example, the combinations 3:3:4:24:1 and 4:4:5:24:1 often perform well. In general, good scoring schemes have slightly lower transition costs than transversion costs, but not so much lower as in the HOXD matrices. They also have high gap existence costs relative to the other scores, compared to the HOXD schemes.

The poor performance of HOXD55:400:30 is not surprising, since these parameters tend to produce alignments with large, random flanks [30]. This explains why it sometimes gives many false positives, but why does it sometimes give few true positives? This is because the score cutoff corresponding to an E-value of 1 is high, e.g. much higher than for HOXD70:400:30 (Additional file 2). We note that many genome alignments in the UCSC database, including human/chicken and human/pufferfish, have been made using HOXD55:400:30. Our results suggest that those alignments could be improved by using different score parameters.

The HOXD55 matrix itself would no doubt perform better if combined with higher gap costs. Indeed, the parameter combination 4:1:4:28:1, which approximates HOXD55 with a large gap opening cost, produces more accurate alignments (Additional file 1, Figure S2, S4). This combination is still mediocre in most tests, however: the best results are obtained using higher transition costs.

**Figure 5:**
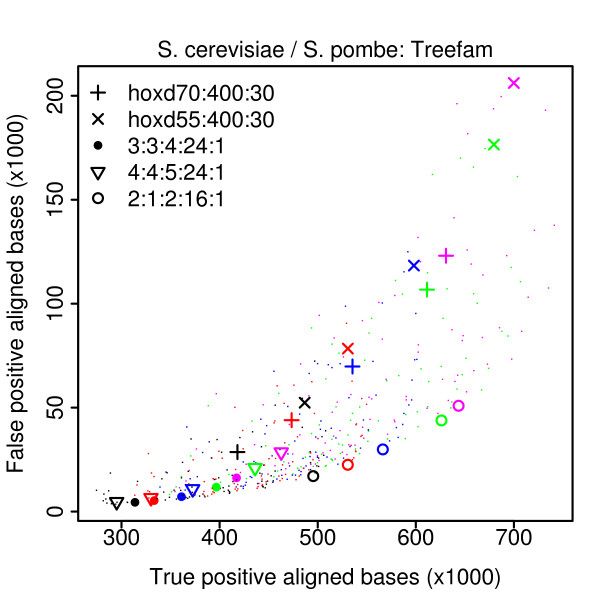
**Genome alignment accuracies using BLASTZ**. This is the same as the lower right panel in Figure 4, except that here the alignments were done with BLASTZ instead of LAST.

To confirm that our results are not specific to LAST, we repeated the assessment for the yeast genomes using BLASTZ (Figure 5). The results are very similar to those using LAST (Figure 4 lower-right), supporting the generality of our conclusions. One difference is that the BLASTZ alignments have somewhat fewer true positives and false positives. This might be because we ran LAST with a spaced seed that is sensitive to protein-coding sequence (Methods). (Although BLASTZ uses spaced seeds, it has no option to use this particular seed.) Both BLASTZ and LAST have algorithmic options that we did not explore, however, so we cannot draw general conclusions from this difference.

### Larger X-drop values are not always better

**Figure 6:**
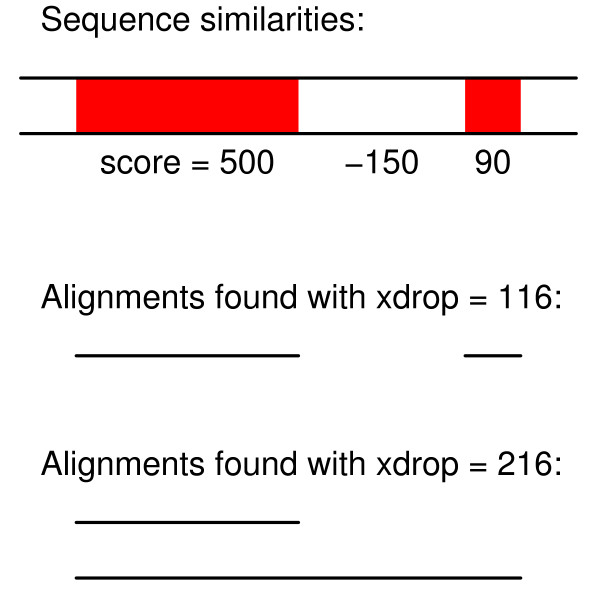
**A problem with large X-drop values**. This sketch represents two similar regions with positive alignment scores (red) separated by a dissimilar region with a negative alignment score. For low X-drop values, the two similar regions are found as separate alignments. For high X-drop values, the X-drop algorithm crosses the dissimilar region: so the alignment seeded from the right-hand similarity has sub-optimal score. In this case, LAST would only report the one alignment with score = 500.

As the X-drop parameter increases, both true positives and false positives usually increase (Figure 4, 5). This is as expected, since larger X-drop values make the algorithm try to extend alignments more aggressively. Surprisingly, however, large X-drop values sometimes lead to fewer true positives and/or false-positives. For example, in several panels of Figure 4, the parameter combination 2:1:2:16:1 produces more true positives with X = 116 (green circles) than with X = 216 (magenta circles). The likely explanation is sketched in Figure 6. When X is large, the alignment can extend over a dissimilar region with large negative score, producing an alignment with a negative-scoring flank [15]. LAST discards such alignments, which can cause it to align fewer bases when X is larger. We do not know exactly how BLASTZ works, but we make two empirical observations: (i) it never seems to align fewer bases when X increases; (ii) it does sometimes produce alignments with negative scoring flanks. NCBI BLAST also exhibits the latter behavior (Additional file 1, Figure S6) [15].

These observations caution against X-drop values much greater than the alignment score cutoff. Above this value, the X-drop algorithm starts to merge alignments that can otherwise be found separately (Figure 6). Using the alignment score cutoff as the X-drop value, often corresponds to a maximum gap size of about 50-100 (Additional file 2), which is fortunately short enough that the gapped extension does not dominate LAST's run time (Table 3 and Additional file 1, Figure S7).

**Table 3:** Run times for LAST genome alignment

Genomes	Index Construction	Alignment
*G. gallus*/*H. sapiens*	11 min	3.5 hrs
*T. rupripes*/*H. sapiens*	3.0 min	2.9 hrs
*A. thaliana*/*O. sativa*	51 sec	38 min
*S. cerevisiae*/*S. pombe*	5.8 sec	1.6 min

CPU time measured for one core of a 3 GHz Xeon E5450 processor is shown. Each alignment time is the median among 495 alignments.

### Problems with scoring matrix evaluation by chaining

Our results seem to conflict with those of Chiaromonte et al. [8]. In our tests the scoring scheme 1:1:1:7:1 performed rather well, whereas in their gapless tests 1:1:1:∞:∞ performed poorly. Chiaromonte et al. used the following evaluation procedure: they aligned human and mouse sequences thought to have evolved without rearrangements, counted aligned bases in the maximal co-linear chain as "correct", and the rest as "incorrect". The human sequence was first masked using RepeatMasker. We replicated their procedure, and confirmed that 1:1:1:∞:∞ performs worse than HOXD70:∞:∞ at all score cutoffs (Additional file 1, Figure S8A). However, most of the high-scoring "incorrect" matches are tandem repeats that were missed by RepeatMasker. So we additionally applied TRF to both sequences. This gives a mixed picture: 1:1:1:∞:∞ performs better than HOXD70:∞:∞ for high score cutoffs, but worse for low score cutoffs (Additional file 1, Figure S8B). The advantage of HOXD70 at low cutoffs comes mainly from its finding longer "correct" alignments, rather than more "correct" alignments (Additional file 1, Figure S8C). These extended alignments are not always truly correct: an example that is surely incorrect is shown in Additional file 1, Figure S8D. Even in less clear-cut cases, the extensions have weak similarity and thus uncertain homology. So the fundamental problem is that co-linear matches need not be correct. In summary, this evaluation procedure based on chaining is not reliable (although the problems were not at all obvious to us beforehand).

### Reliable pairings found by alignment probabilities

An obvious and useful application of alignment probabilities is to annotate alignments with column reliability estimates. These probabilities can also be used, however, to compute pair-wise alignments that are expected to contain more correct base pairs than standard maximum-score alignments. Several methods have been proposed for making alignments using such probability estimates, including: centroid alignment [18,31], MPD alignment [16], and AMA alignment [32]. All of these methods can benefit from an appropriate choice of alignment parameters. Here we used γ-centroid alignments [33], which maximize the expected value of: γ TP + TN, where TP is the number of true positive aligned bases, TN is the number of true negative aligned bases, and γ is a user defined parameter to control the tradeoff between sensitivity and specificity. This optimization function fits well with the criteria we used for evaluating alignment quality. When γ = 1 it is equivalent to centroid alignment [18], and when γ = ∞ it is equivalent to the alignment method described in [34]. When γ ≤ 1, γ-centroid alignment is especially simple: it just aligns all pairs of bases that have alignment probability greater than 1/(1+γ).

**Figure 7:**
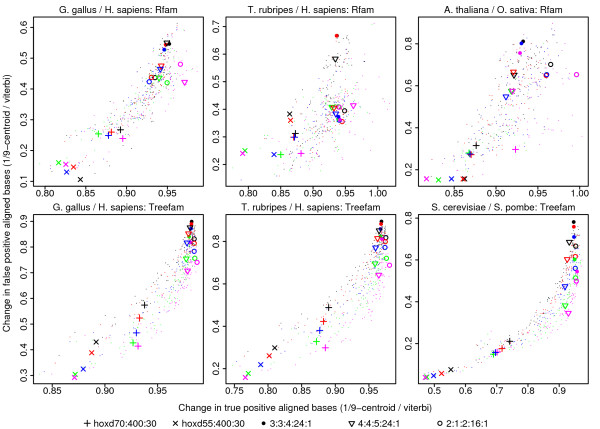
**Genome alignment accuracies for 1/9-centroid alignment compared to ordinary (Viterbi) alignment**. Each point represents one combination of score parameters. A few of these are highlighted with symbols: see the key beneath the figure. Colors indicate the X-drop parameter as in Figure 4. The X-coordinate indicates the number of true positives for 1/9-centroid alignment as a fraction of the number of true positives for Viterbi alignment. Likewise for the Y-coordinate and false positives. The 1/9-centroid results alone, without comparison to Viterbi alignment, are shown in Additional file 1, Figure S9.

We attempted to find highly reliable aligned bases by performing γ-centroid alignment with γ = 1/9. This means that we aligned all pairs of bases whose estimated probability of aligning is greater than 90% [33]. Figure 7 shows the change in true positive and false positive aligned bases, compared to standard maximum-score alignment (Figure 4). In general, true positives decrease only slightly, but false positives decrease greatly. Thus, we do indeed obtain more reliable pairings, with only a modest sacrifice of sensitivity. Interestingly, the effect is more dramatic for scoring schemes that perform badly with standard maximum-score alignment, such as the HOXD scoring schemes. Therefore, the performance of the HOXD schemes catches up with, and sometimes surpasses, that of schemes such as 2:1:2:16:1 (Additional file 1, Figure S9).

We also investigated centroid alignment (i.e. γ-centroid alignment with γ = 1). This means that we aligned all pairs of bases whose estimated probability of aligning is greater than 50% [18,31]. Generally, sensitivity (true positives) changes very little compared to standard maximum-score alignment, while false positives sometimes decrease substantially (Additional file 1, Figure S10). The improvement may not be sufficient to justify using an exotic alignment method.

## Discussion

Taken together, these results allow us to align genomes with greater accuracy and better estimates of the error rates.

### Repeat masking

We have shown that standard E-value calculations predict the rate of spurious alignment quite accurately, if tandem repeats are carefully masked. It is interesting that masking tandem repeats is sufficient: this suggests that simple (low entropy) sequences other than tandem repeats are rare. Our TRF masking protocol leaves room for improvement, e.g. it works less well than DustMasker for Arabidopsis/Rice (Figure 1). Nevertheless, it suppressed *all *spurious alignments with E-value less than 10^-4 ^(Figure 1, S1): a powerful result.

### Score parameters

The scoring scheme 2:1:2:16:1 gives very accurate alignments of distantly-related protein-coding sequences, whereas 1:1:1:7:1 is a good, conservative all-round choice. The main difference from the UCSC HOXD schemes is a relatively larger gap existence cost. Kent et al. used high gap existence costs when aligning nematode genomes [35]. Lunter found high indel rates for human/mouse [36], which could be taken to suggest that gap existence costs should be low, but with simulated data they also found that lowering gap costs to make the gap frequency of alignment match the true gap frequency did not help - "the number of gaps can be made to approximate the true indel count, but only at the expense of placing the gaps in the wrong positions" [16]. A secondary reason for poor performance of the HOXD schemes may be overly weak transition penalties compared to the matrices in Table 2.

Since our assessment of score parameters is restricted to protein- and RNA-coding regions, there remains much uncertainty about these parameters for genome alignment in general. We can at least say this: there is both statistical and now (limited) empirical evidence that HOXD55:400:30 is a poor choice, and, ironically, there is little reason to reject the simplest possible (+1/-1) scoring matrix.

### Range of parameter search

The scoring schemes tested here use small integers, compared to the HOXD matrices. Small integers have some practical advantages: they work better with several methods for calculating statistical parameters [30,37,38], and they reduce the risk of computer overflow when comparing a genome to itself. Besides, our ability to discriminate effective parameters does not justify more than one significant figure.

Another constraint we applied to our parameter search is that all matches were given the same score. One might expect better performance by tuning the match scores to reflect A+T content. However, to be effective, this adjustment must consider not only the overall A+T content of the concerned genomes, but also the A+T content of the target homologous sequences. Note that for many genomes the A+T content of the Rfam sequences exceeds 50%, while the A+T content of the TreeFam sequences falls below the 50% level (Table 2 and Additional file 1, Table S2). Thus for the genomes studied, we believe that it is a reasonable compromise to treat all matches equally. We leave for future work the consideration of score parameters for highly A+T skewed genomes, such as that of the malaria parasite *Plasmodium falciparum*.

### Local versus global alignment

Our conclusions concerning alignment parameters may not apply to global alignment, where a whole pre-defined sequence is forced to align.

Some genome alignment methods have aspects of global alignment. BLASTZ has an option to perform high-sensitivity local alignment between adjacent "anchoring" local alignments. Some other methods force global alignment between such anchors: the motivation is presumably to increase sensitivity, but this may also decrease specificity, especially if dissimilar sequences are forced to align. Another common approach is to find chains of co-linear local alignments: the hope may be to increase sensitivity by lowering the alignment score cutoff, while avoiding spurious alignments by the chaining requirement. All these approaches are intuitively reasonable, because they reflect a prior expectation of finding co-linear homologies. On the other hand, they obviously introduce a bias towards finding co-linear similarities, which might be best avoided when studying genome rearrangement history, for example. In summary, local alignment is a conservative approach with well-understood statistics, whereas the more global methods are more aggressive.

### Relevance to more complex alignment methods

This study focuses on classic Smith-Waterman type pair-wise alignment (with X-drop) and affine gap penalties. Although more complex methods are available (e.g. [16,35,39-41]), widely used genome alignments are still based on classic alignment [7], and so our results have practical relevance. In general, the more complex methods seem to differ from classic alignment in three ways:

i) They use explicit probabilistic models.

ii) They use probabilistic algorithms such as posterior decoding, rather than Viterbi/maximum-score alignment.

iii) They use more intricate models with more parameters.

The first is not really a difference, because classic alignment is equivalent to Viterbi decoding with a probabilistic model (a pair hidden Markov model) [12]. As for the second, previous studies have shown that probabilistic algorithms can be more accurate than maximum-score alignment [16,18]. In our results, a probabilistic algorithm (γ-centroid alignment) improved accuracy for bad score parameters such as HOXD55:400:30, but the benefit is not so clear for good score parameters (Figure 7 and Additional file 1, Figure S10). This may be because the good parameters have high enough gap costs that there is not much uncertainty in the alignments. Nevertheless, alignment probabilities are clearly useful for indicating the confidence that each pair of bases is homologous. Finally, the benefit of intricate modeling is unclear: Lunter et al. found only modest improvements, even though their test data consisted of simulations from their model [16]. They concluded that use of alignment probabilities is more important than model accuracy.

One limitation of these more complex alignment methods is that they seem to fit more easily with global than local alignment. In particular, it is unclear how to calculate E-values that discriminate (local) homologies from chance similarities. It is also not obvious how to adapt their parameter-training algorithms to local alignment: we tried to do this, with poor results (not shown). In fact, many of these methods use BLASTZ alignments as a starting point, so they directly depend on accurate classic alignments. We expect that both simple and complex genome alignment methods will be useful in future, as is the case for protein alignment.

Some sophisticated methods attempt not merely pair-wise but multiple genome alignment [39,41,42]. This is a much harder problem. The only comment we make is that multiple genome alignments are always built from pair-wise alignments in one way or another, and thus accurate pair-wise alignment is beneficial for accurate multiple alignment.

Finally, parametric alignment [43] has been suggested as a useful technique for genome alignment score parameter selection [44]. Parametric alignment computes the set of maximum likelihood alignments obtained by all possible settings of the score parameters. More to the point, parametric alignment also provides a finite (but sometimes large) set of parameter settings guaranteed to cover all alignments which can be optimal for any parameter setting, and thus can guide an efficient search for a parameter setting which maximizes some desired alignment quality. Unfortunately, practical parametric alignment techniques have only been developed for global alignment. Indeed Dewey et al. first used a local alignment based technique to identify similar segments and only applied parametric alignment to the resulting segments. In any case, they did not make specific recommendations for appropriate parameter settings. A related approach, inverse parametric alignment [45,46], finds parameters that cause given example alignments to have near-optimal scores. This approach was found to improve global alignment of proteins, but its efficacy for local alignment of DNA has yet to be tested.

## Conclusions

We have conducted the first large-scale assessment of repeat masking strategy and genome alignment parameters using real genomes - producing a practical guide to alignment parameters. We have tested a sufficient number of parameter combinations and genome pairs so that our results will be relevant to most genome alignment tasks. With our results, researchers will not only be able to produce more accurate alignments than with previous standard practice, but they will also have a much better idea of the reliability they can expect from such alignments.

## Methods

### Genome data

The following genome versions were obtained from UCSC http://genome.ucsc.edu/: hg18, galGal3, fr2, gasAcu1, ce6, caePb2 [7]. The remaining genomes were obtained from RefSeq [47]. *E. coli*: NC_000913.2. *B. subtilis*: NC_000964.2. *S. pombe*: NC_001326.1, NC_003421.2, NC_003423.3, NC_003424.3. *A. thaliana*: NC_003074.5, NC_003071.4, NC_003070.6, NC_003075.4, NC_003076.5. *O. sativa*: NC_008394.1, NC_008395.1, NC_008396.1, NC_008397.1, NC_008398.1, NC_008399.1, NC_008400.1, NC_008401.1, NC_008402.1, NC_008403.1, NC_008404.1, NC_008405.1. *S. cerevisiae*: NC_001133.7, NC_001134.7, NC_001135.4, NC_001136.8, NC_001137.2, NC_001138.4, NC_001139.8, NC_001140.5, NC_001141.1, NC_001142.7, NC_001143.7, NC_001144.4, NC_001145.2, NC_001146.6, NC_001147.5, NC_001148.3, NC_001224.1. For the chaining evaluation, we obtained human and mouse CD4 regions from GenBank: U47924.1 and AC002397.1.

### E-value calculations

E-values are related to alignment scores by the following equation: E-value = 2 mnK * exp(-λ * score) [9]. Here, m and n are the lengths of the two genomes, and the factor of 2 appears because we compare both strands. We defined genome length as the total count of A, C, G, and T (i.e. ambiguous bases aren't counted) before any repeat-masking. The parameters λ and K depend on the scoring matrix, gap costs, and base abundances. We calculated them using ALP 1.1 http://www.ncbi.nlm.nih.gov/CBBresearch/Spouge/html.ncbi/index/software.html with options: -eps_lambda 0.01 -eps_K 0.05 -max_time 600 -max_mem 2000[38]. For the base abundances, we used the average of the two genomes (i.e. we obtained the percentages for each genome separately, and then averaged those percentages).

### Repeat-masking

For all alignments, any repeat masking was applied equally to both genomes. We ran Tandem Repeats Finder 4.04 with these options: match = 2 mismatch = 5 delta = 5 PM = 80 PI = 10 minscore = 30 maxperiod = 200 -R. For "TRFs" (Figure 1), we used: match = 2 mismatch = 7 delta = 7 PM = 80 PI = 10 minscore = 50 maxperiod = 2000. For DustMasker, we used the option "-level 16", which increases the amount of masking. Runnseg has trouble with large runs of "N" in sequences: so we split sequences at runs of 100 or more "N"s, applied runnseg to the fragments, and then re-joined them.

Figures 1 and 3 show alignments with twenty combinations of score parameters: 1:1:1:{2,5}:1, 2:{1,2}:2:{5,8,12}:1, and {HOXD55, HOXD70}:400:30, with the X-drop parameter set to allow a maximum gap size of either 20 or 50.

For the chaining evaluation, we used RepeatMasker open-3.2.7 (default mode) with cross_match = 0.990329, RepBase Update 20090120, and RM database version 20090120. In this case only, we masked just one sequence (human), for consistency with Chiaromonte et al. [8].

### Gold-standard alignments

We used the alignment files Rfam.full from Rfam 9.0 and aa_full_align from TreeFam 6.0 [28,29]. We excluded some TreeFam proteins whose lengths were not consistent between aa_full_align and aa_seq. We then mapped the sequences to their parent genomes using BLAT version 34, with -ooc = 11.ooc for Rfam, and -q = prot -t = dnax -tileSize = 8 for TreeFam [48]. We kept BLAT alignments where the number of identical matches equaled the query sequence length (i.e. we required perfect identity apart from introns). Finally, we used just one mapping for each TreeFam and Rfam family (the first in the genome, with chromosomes in ASCII-betical order), to avoid double counting from non-independent alignments. The BLAT mappings, together with the Rfam and TreeFam alignments, define partial genome-to-genome alignments. The number of aligned bases in each gold-standard is shown in Additional file 1, Table S3.

True positives are defined to be aligned bases in the LAST (or BLASTZ) genome alignment that are also aligned to each other according to the gold-standard. For Rfam, false positives are defined to be aligned bases in the LAST (or BLASTZ) genome alignment, that are both aligned according to the gold-standard, but not to each other. (So if a base is not aligned at all in the gold-standard, an alignment of this base is not counted as a false positive nor as a true positive. This is reasonable because the gold-standard is very incomplete.) For TreeFam, we defined false positives more narrowly: we only counted aligned bases from the same TreeFam family. This is because there are inter-family homologies that are not captured in the TreeFam alignments.

We also performed some tests using the curated TreeFam file aa_seed_align instead of aa_full_align: the results are very similar (Additional file 1, Figure S5).

### LAST

We intend to describe LAST in detail elsewhere: here is a minimal description. LAST follows the same three steps as BLAST and BLASTZ: find initial matches, extend them using a gapless X-drop algorithm, and finally extend them using a gapped X-drop algorithm [6,14]. The main difference lies in how it finds initial matches. Whereas BLASTZ finds fixed-length matches (e.g. 12-mers), LAST finds variable-length matches: specifically, it finds all matches, of any size, that occur no more than ten times in the target genome. (One of the two genomes is arbitrarily designated as the "target": in this study, the first-named genome is always the target.) The motivation is to avoid an excessive number of initial matches due to non-uniform sequence composition. (Repeat-masking only partially solves this problem.) LAST is freely available at http://last.cbrc.jp/, or in Additional file 3.

LAST has an option to estimate alignment probabilities, as follows. It first performs standard gapped X-drop extensions in both directions from a "seed". Each X-drop extension defines a limited area of the dynamic programming matrix [14]. LAST then calculates probabilities within this area by applying a forward-backward algorithm [12]. It assumes that each gapped extension has probability proportional to exp(λ * score), where λ is the implicit scale factor of the scoring matrix [49]. It also assumes that the seed is correctly aligned with probability 1.0, so that one of the possible gapped extensions in each direction is definitely correct. In other words, the alignment probabilities are conditional on the alignment not being wholly spurious, which must be remembered when interpreting them. (Since the seed is in a highly similar part of the alignment, it is likely to be correctly aligned provided the alignment reflects a true homology.)

We ran lastdb (to construct an index of the target genome) with the following parameters: -c -m110 -s5G. "-m110" tells it to ignore every third base when finding initial matches: this makes it considerably more sensitive for protein-coding sequence, and somewhat more sensitive for noncoding sequence (compared to contiguous seeds). We ran lastal (to do the alignments) with options -p, -a, -b, -e, and -x to set the score parameters. For soft-masking, we used lastal option -u to mask during gapless but not gapped extension. Finally, we set the gapless X-drop parameter (lastal -y) to: 10 * (maximum value in the scoring matrix).

### BLASTZ

We ran BLASTZ v7 with these options: T = 2 M = 50. We also used options Q, O, E, L, and Y to set the score parameters. Finally, we set the gapless score cutoff (K) to L*3/5, for consistency with LAST. In the right-most panel in row 6 of Figure 1, six of the twenty scoring schemes are omitted, because these BLASTZ runs did not finish even after hundreds of hours. For the chaining evaluation, we ran BLASTZ with P = 0 and either C = 3 (no chaining) or C = 1 (chaining). We used eleven score cutoffs (K): 20 to 30 for the unit matrix, and 2000 to 3000 for HOXD70.

## Authors' contributions

MCF conceived of the study, performed the experiments, and helped to draft the manuscript. MH developed the probabilistic alignment methods and helped with the relevant sections of the manuscript. PH developed prototypes of LAST and helped to draft the manuscript. All authors read and approved the final manuscript.

## Supplementary Material

Additional file 1 **Supplementary document**. Three tables and nine figures. Click here for file

Additional file 2 **Supplementary data**. Score parameter combinations, their statistical parameters λ and K, and alignment accuracy results. Click here for file

Additional file 3 **LAST version 94**. Source code and documentation. Click here for file
