# Supplementary material for: Parameters for accurate genome alignment
Source: BMC Bioinformatics. 2010 Feb 9;11:80. doi: 10.1186/1471-2105-11-80 (PMC2829014; doi:10.1186/1471-2105-11-80)
Supplement: Additional file 1 — Supplementary document. Three tables and nine figures. [file 1471-2105-11-80-S1.PDF]

Supplementary Material for

“Parameters for accurate genome alignment”

by Martin C. Frith, Michiaki Hamada, and Paul Horton

Table S1: Parameters that have been used for some pair-wise genome alignments in the UCSC genome database

Genomes	Score parameters	Alignment score cutoff	E-value
G. gallus (galGal3) / *H. sapiens* (hg18)	HOXD55:400:30	L=10000	5e-10
T. rubripes (fr2) / *H. sapiens* (hg18)	HOXD55:400:30	L=6000	0.9
D. melanogaster (dm3) / *D. yakuba* (droYak2)	HOXD55:400:30	L=4000	9000
D. melanogaster (dm3) / *D. ananassae* (droAna3)	HOXD55:400:30	L=4000	14000
T. rubripes (fr2) / *G. aculeatus* (gasAcu1)	HOXD55:400:30	L=6000	0.05
C. elegans (ce6) / *C. briggsae* (cb3)	HOXD70:400:30	L=3000	7000

"HOXD55:400:30" means: the HOXD55 scoring matrix with a gap existence cost of 400 and a gap extension cost of 30. "L" is the name of BLASTZ's alignment score cutoff option (for gapped alignments).

Table S2: Log-likelihood-ratio scoring matrices

Dm/Dy Rfam

91% identity, 56% A+T

	a	c	g	t
a	84	-172	-125	-151
c	-172	100	-169	-125
g	-125	-169	100	-172
t	-151	-125	-172	84

Dm/Da Rfam

80% identity, 57% A+T

	a	c	g	t
a	78	-110	-84	-94
c	-110	100	-131	-84
g	-84	-131	100	-110
t	-94	-84	-110	78

Tr/Ga Rfam

77% identity, 51% A+T

	a	c	g	T
a	94	-123	-75	-106
c	-123	100	-137	-75
g	-75	-137	100	-123
t	-106	-75	-123	94

Dm/Dy TreeFam

88% identity, 45% A+T

	a	c	g	t
a	100	-156	-107	-154
c	-156	88	-158	-107
g	-107	-158	88	-156
t	-154	-107	-156	100

Dm/Da TreeFam

73% identity, 45% A+T

	a	c	g	t
a	100	-105	-59	-115
c	-105	85	-101	-59
g	-59	-101	85	-105
t	-115	-59	-105	100

Ce/Cb TreeFam

69% identity, 55% A+T

	a	c	g	t
a	85	-98	-51	-98
c	-98	100	-106	-51
g	-51	-106	100	-98
t	-98	-51	-98	85

Score matrices derived from Rfam (top row) and TreeFam (bottom row) based gold standards for the indicated genome pairs are shown. Species are names abbreviated as: Dm: *D. melanogaster*, Dy: *D. yakuba*, Da: *D. ananassae*, Tr: *T. rubripes*, Gg: *G. gallus*, Ce: *C. elegans*, Cb: *C. briggsae*. Table 2 shows corresponding matrices for other genome pairs.

Table S3: How many pairs of aligned bases in each gold-standard genome alignment

Genomes	Rfam	TreeFam "full" (TreeFam-B)	TreeFam "seed" (TreeFam-A)
G. gallus / H. sapiens	18373	3971076	354675
T. rubripes / H. sapiens	10838	3995469	338481
A. thaliana / O. sativa	7324		
S. cerevisiae / S. pombe		1825374	
D. melanogaster / D. yakuba	7881	6609996	
D. melanogaster / D. ananassae	7300	6053292	
T. rubripes / G. aculeatus	10238		406137
C. elegans / C. briggsae		3518235	

Figure S1: E-values of mammalian reverse genome alignments with tandem repeat masking

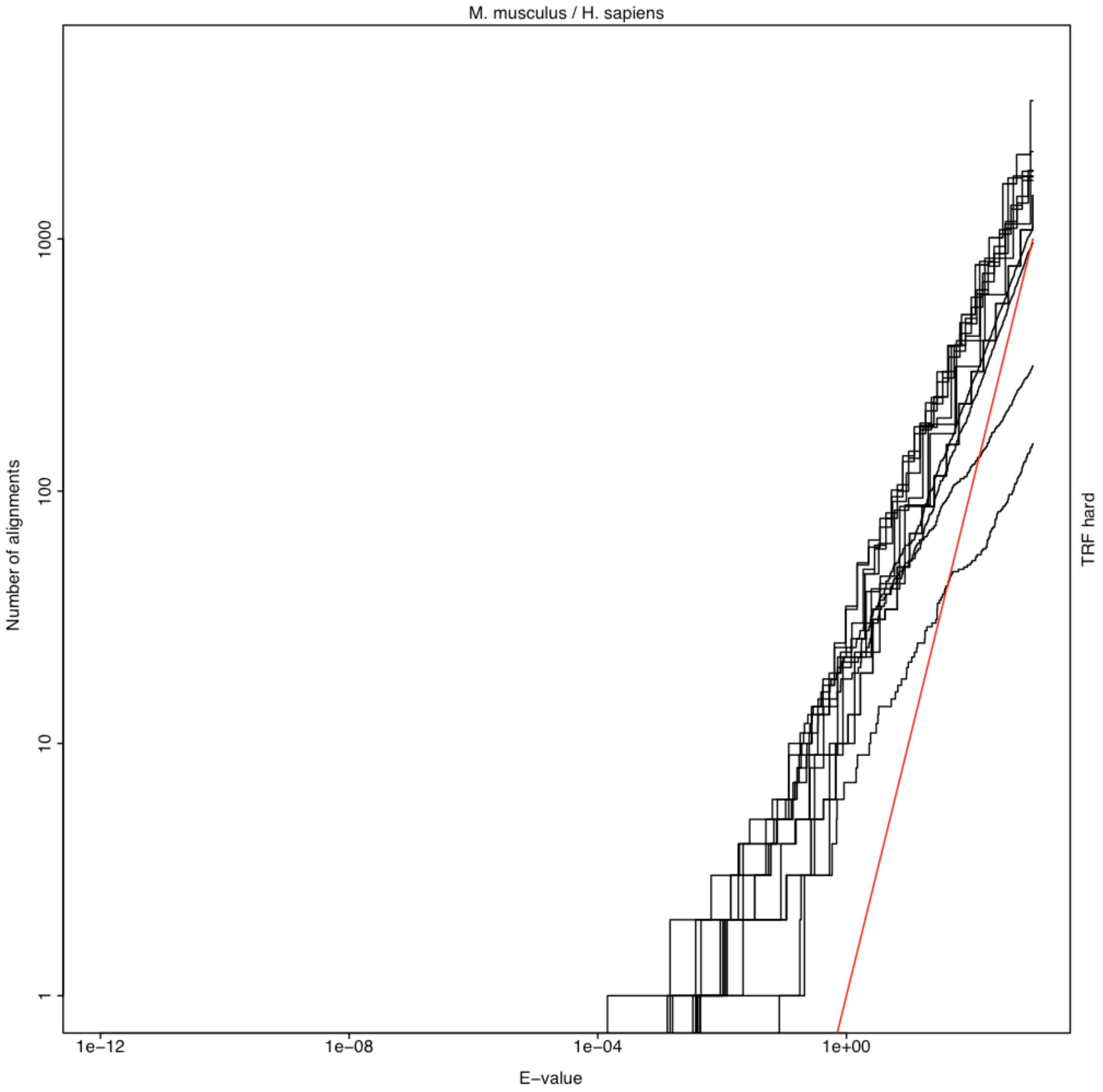

The human genome was reversed (but not complemented) and then aligned to the mouse genome twenty times, using twenty different scoring schemes. The red line shows the theoretically expected number of alignments at each E-value threshold, and the black lines show the observed number. There are twenty black lines. The alignments were performed with LAST. Both genomes were hard-masked using Tandem Repeats Finder version 4.04, with these settings: `match=2 mismatch=5 delta=5 PM=80 PI=10 minscore=30 maxperiod=200 -R`.

Figure S2: Genome alignment accuracies with 495 combinations of score parameters

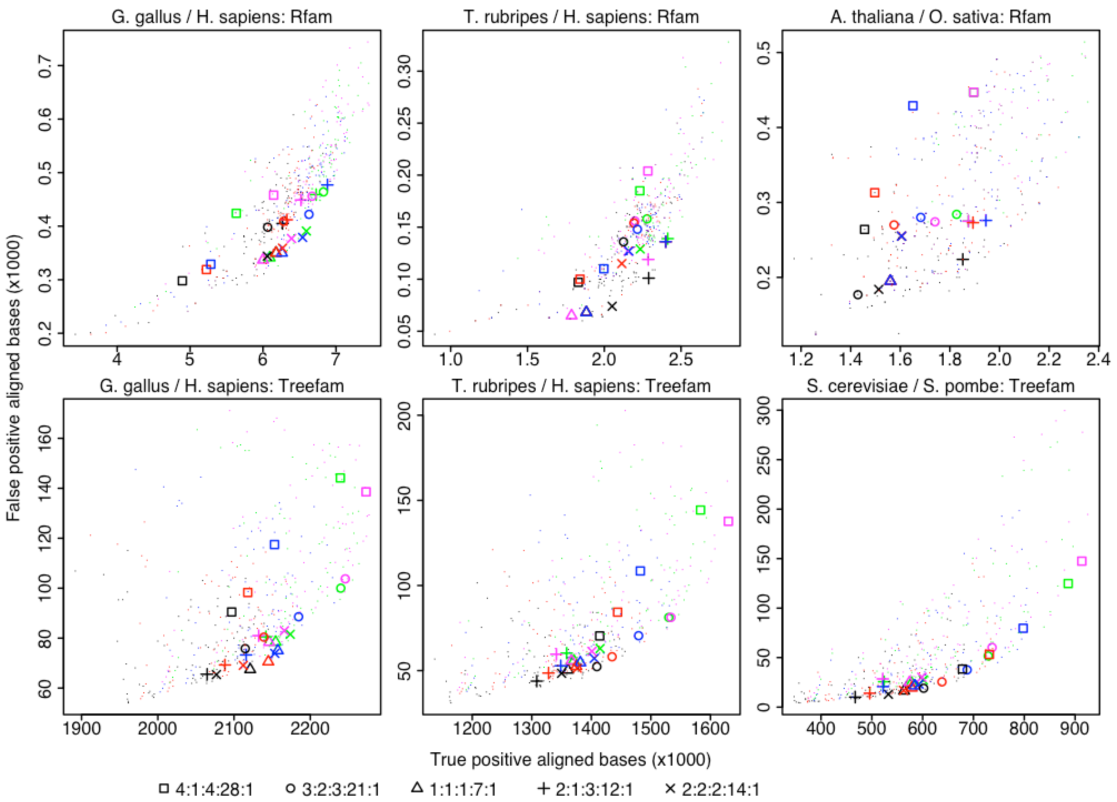

Each point represents one genome alignment with one combination of score parameters. A few of these are highlighted with symbols: see the key beneath the figure. True positives and false positives were counted with reference to either TreeFam or Rfam as indicated. Colors indicate the X-drop parameter. For black points: the X-drop parameter was set so as to allow a maximum gap size of 20; red: 30; blue: 50; green: 100 and magenta: 200. The same results, but with different scores highlighted are shown in Figure 4. Figures S3 and S4 are similar plots using different genomes.

Figure S3: Genome alignment accuracies with 495 combinations of score parameters

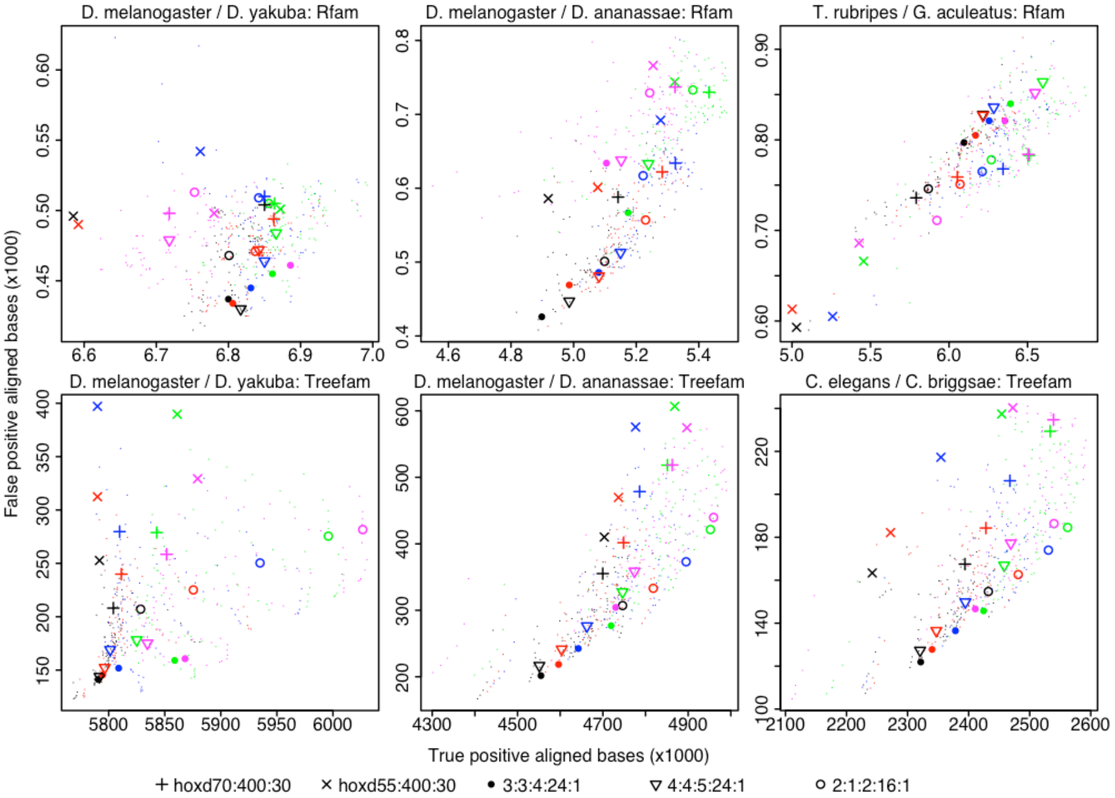

Each point represents one genome alignment with one combination of score parameters. A few of these are highlighted with symbols: see the key beneath the figure. True positives and false positives were counted with reference to either TreeFam or Rfam as indicated. Colors indicate the X-drop parameter. For black points: the X-drop parameter was set so as to allow a maximum gap size of 20; red: 30; blue: 50; green: 100 and magenta: 200. The same results, but with different scoring schemes highlighted, are shown in Figure S4. Figures 4 and S2 are similar plots using different genomes.

Figure S4: Genome alignment accuracies with 495 combinations of score parameters

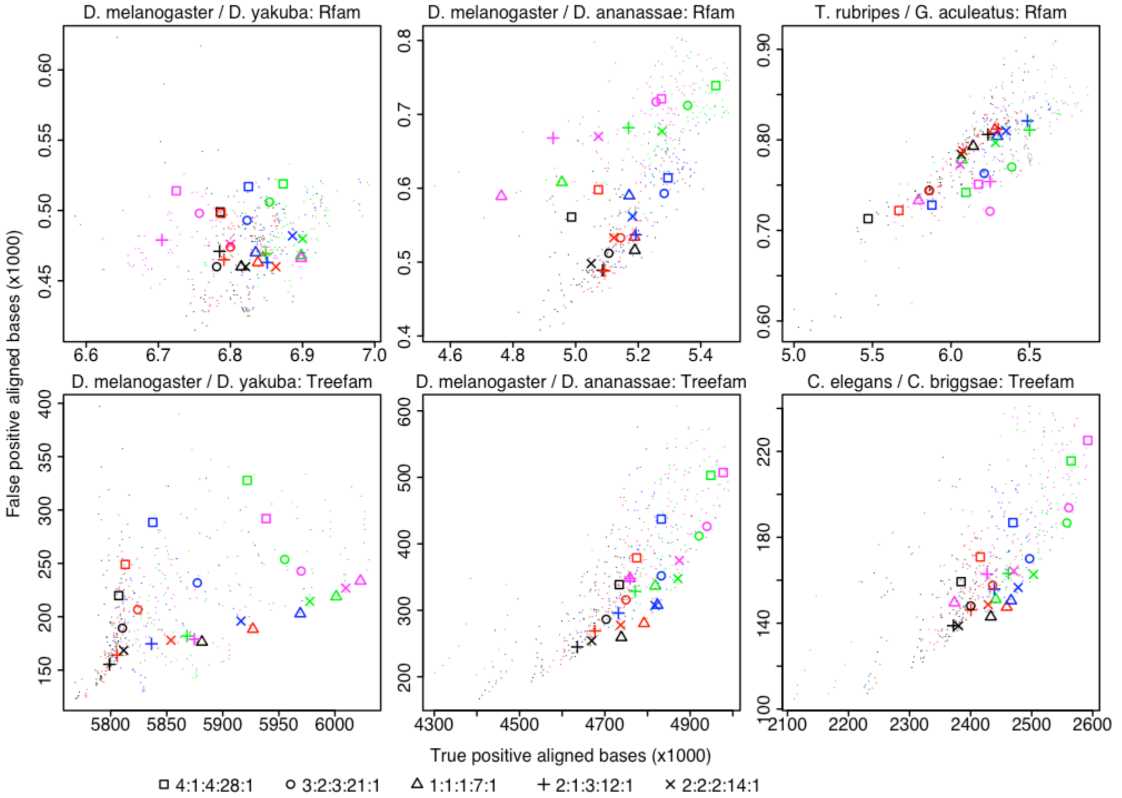

Each point represents one genome alignment with one combination of score parameters. A few of these are highlighted with symbols: see the key beneath the figure. True positives and false positives were counted with reference to either Treefam or Rfam as indicated. Colors indicate the X-drop parameter. For black points: the X-drop parameter was set so as to allow a maximum gap size of 20; red: 30; blue: 50; green: 100 and magenta: 200. The same results, but with different scoring schemes highlighted, are shown in Figure S3. Figures 4 and S2 are similar plots using different genomes.

Figure S5: Genome alignment accuracies with 495 combinations of score parameters

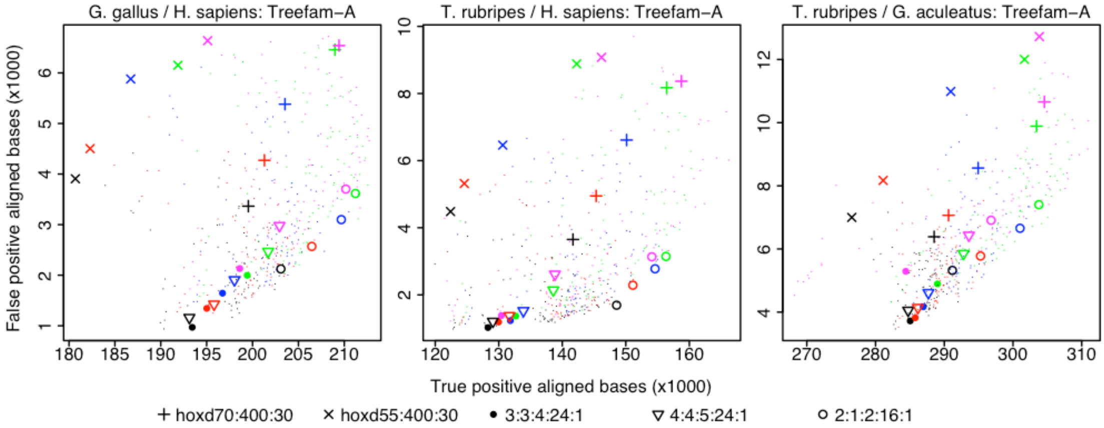

Each point represents one genome alignment with one combination of score parameters. A few of these are highlighted with symbols: see the key beneath the figure. True positives (horizontal axis) and false positives (vertical axis) were counted with reference to TreeFam-A. Colors indicate the X-drop parameter. For black points: the X-drop parameter was set to allow a maximum gap size of 20; red: 30; blue: 50; green: 100 and magenta: 200.

Figure S6: An alignment with negative-scoring flank returned by NCBI BLAST

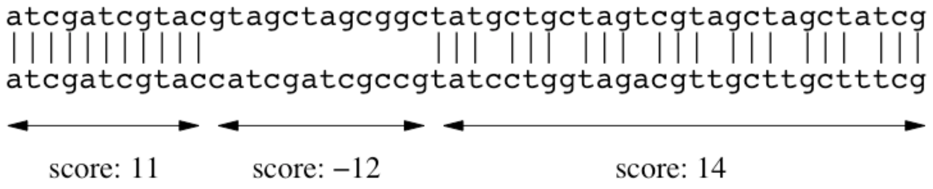

The two depicted sequences were aligned using bl2seq version 2.2.20 with the following options:

`-i topseq.fasta -j botseq.fasta -p blastn -q -1`.

Thus, negative-scoring flanks are not unique to BLASTZ.

Figure S7: Run times for genome alignments with LAST

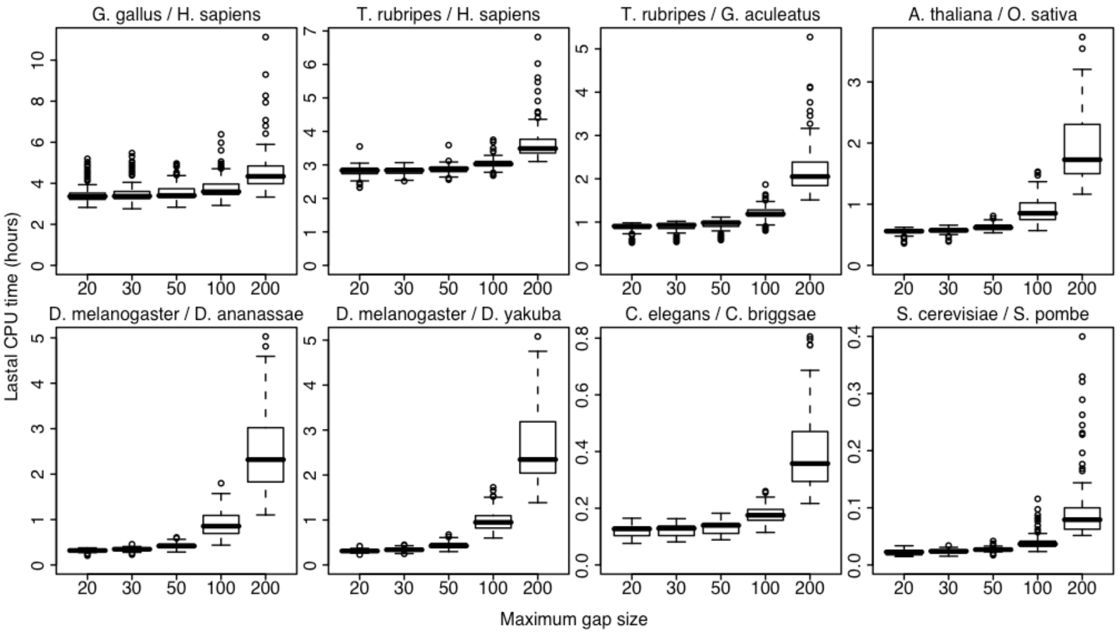

Each panel shows 495 run times for 495 parameter combinations. These are separated into five groups of 99 based on the X-drop parameter. The X-drop parameter was set so as to allow a maximum gap size of 20, 30, 50, 100, or 200. Each genome alignment was performed on one core of a 3GHz Xeon E5450 processor.

Figure S8: Comparing score matrices by chaining gapless alignments

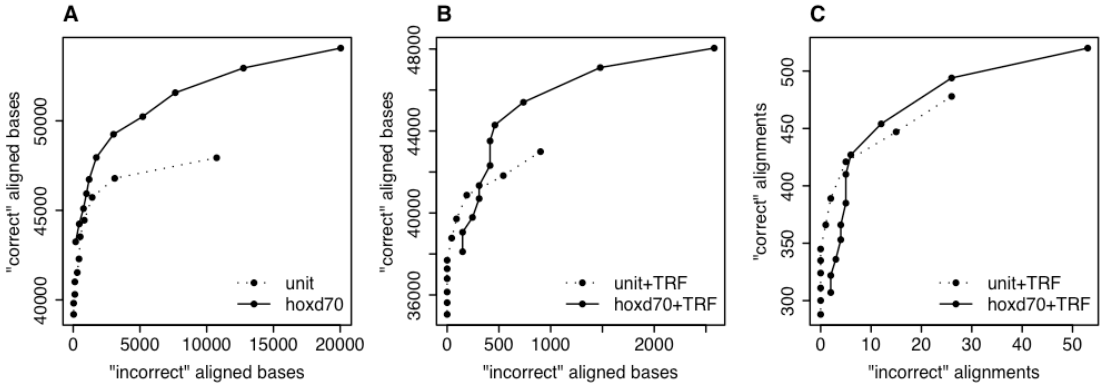

D

Unit+TRF

```
TGTGGGTGTCTGAGGCGAAGAAGAGGATGGCGGAGGTTGCAGCCAC
||| ||   | ||||| ||| ||||| |||| ||||||||||||||
TGTAGGCACCCGAGGCAAAGGAGAGGGTGGCAGAGGTTGCAGCCAC
```

Hoxd70+TRF

```
TGTGGGTGTCTGAGGCGAAGAAGAGGATGGCGGAGGTTGCAGCCACCAACCACAAGAGTTCCTTAGAGGGGTCACA
||| ||   | ||||| ||| ||||| |||| ||||||||||||||  | |   |  | ||  |    ||||||||
TGTAGGCACCCGAGGCAAAGGAGAGGGTGGCAGAGGTTGCAGCCACTGAACCACAAGGGTCGCTTAGAGGGTCACA
```

Hand alignment

```
TGTGGGTGTCTGAGGCGAAGAAGAGGATGGCGGAGGTTGCAGCCACC-AACCACAAGAGTTCCTTAGAGGGGTCACA
||| ||   | ||||| ||| ||||| |||| ||||||||||||||  ||||||||| ||  |||||| ||||||||
TGTAGGCACCCGAGGCAAAGGAGAGGGTGGCAGAGGTTGCAGCCACTGAACCACAAGGGTCGCTTAGA-GGGTCACA
```

The human and mouse CD4 regions were aligned using either the unit matrix (match/mismatch = 1) or the HOXD70 matrix. These alignments were done without allowing gaps, just as in the earlier study by Chiaromonte et al. With each matrix, eleven score cutoffs were tried. Matches appearing in the same relative order in each sequence were classed as "correct", others as "incorrect". **A**: Results after applying RepeatMasker to the human sequence. **B**: Results after further applying TRF to both sequences. **C**: Counts of alignments rather than aligned bases. **D**: An example where HOXD70 finds more chained matches than the unit matrix, but some of them are actually incorrect. The correct alignment has two gaps. (Since the alignment procedure used here does not allow gaps, it is impossible to obtain the correct alignment.) In conclusion, co-linear matches are not necessarily correct, and this is not a reliable way to compare the alignment accuracies achieved by different score matrices.

Figure S9: Genome alignment accuracies for 1/9-centroid alignment

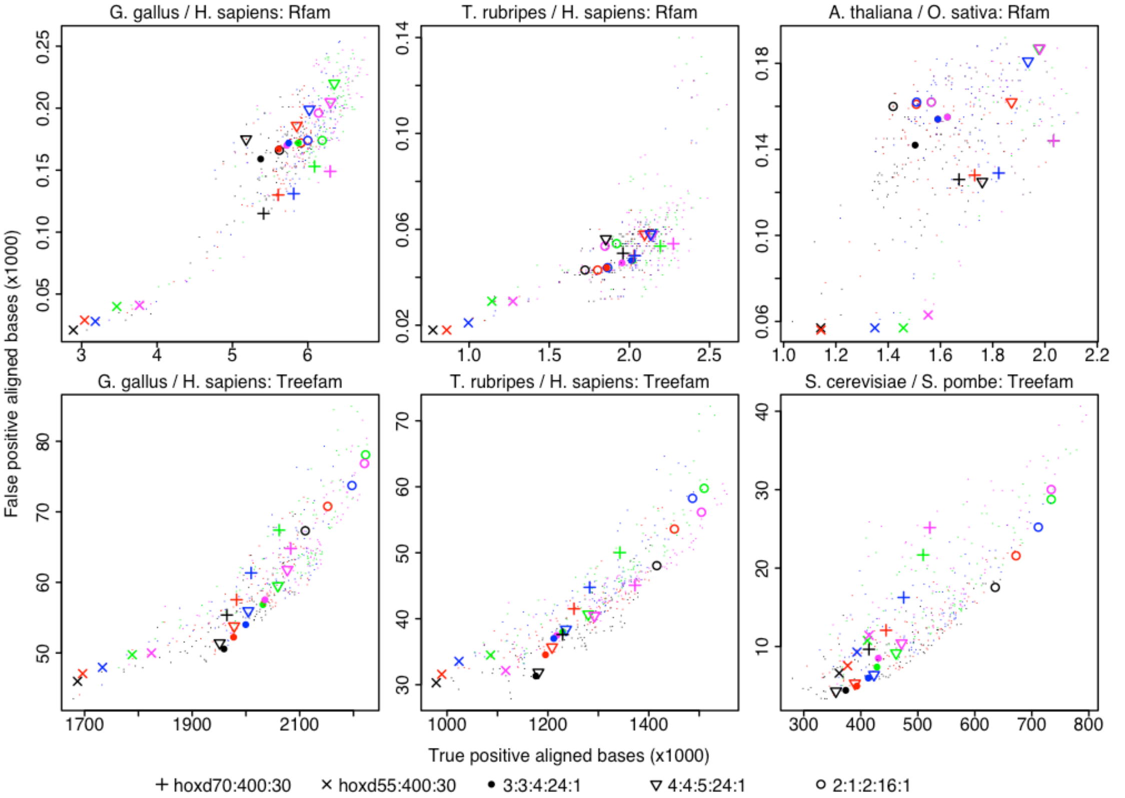

Each point represents one genome alignment with one combination of score parameters. A few of these are highlighted with symbols: see the key beneath the figure. True positives (horizontal axis) and false positives (vertical axis) were counted with reference to either Rfam (top row) or TreeFam (bottom row). Colors indicate the X-drop parameter. For black points: the X-drop parameter was set to allow a maximum gap size of 20; red: 30; blue: 50; green: 100 and magenta: 200. Figure 6 shows the same results, but as fractions of the results obtained using ordinary (Viterbi) alignment.

Figure S10: Genome alignment accuracies for centroid alignment compared to ordinary (Viterbi) alignment

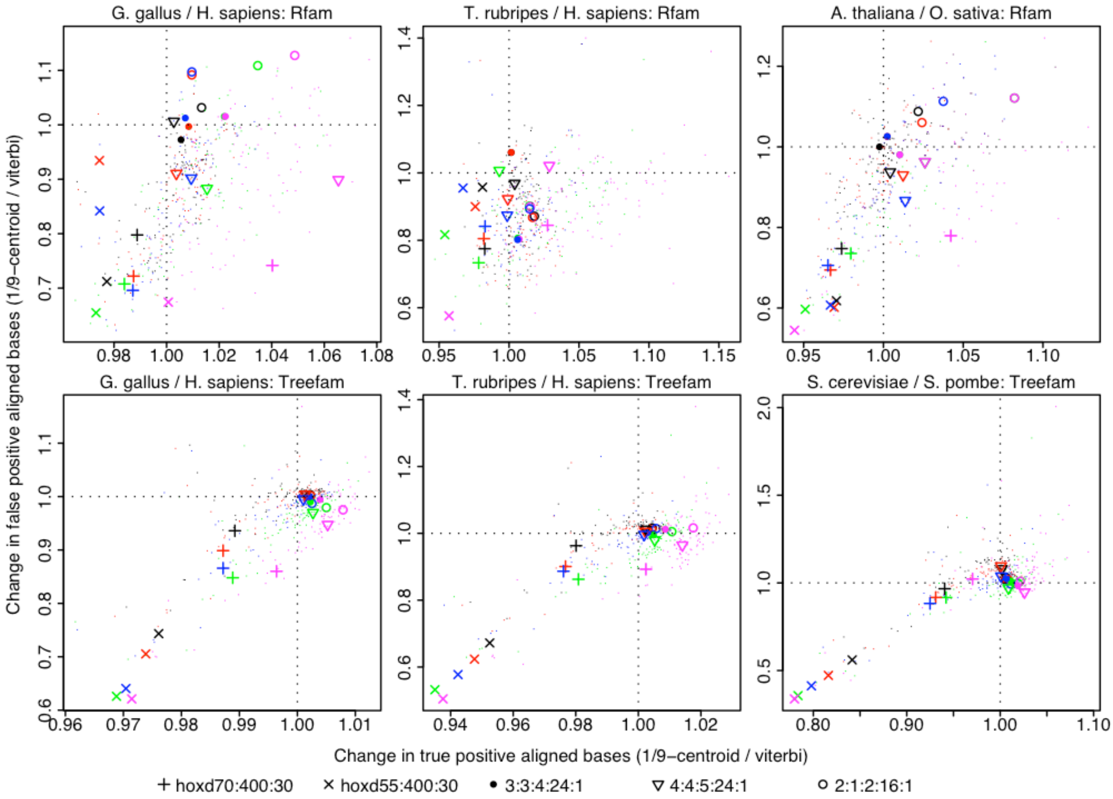

Each point represents one combination of score parameters. A few of these are highlighted with symbols: see the key beneath the figure. Colors indicate the X-drop parameter as in other figures. The X-coordinate indicates the number of true positives for centroid alignment as a fraction of the number of true positives for Viterbi alignment. Likewise for the Y-coordinate and false positives.
